# Analysis of a β-helical region in the p55 domain of *Helicobacter pylori *vacuolating toxin

**DOI:** 10.1186/1471-2180-10-60

**Published:** 2010-02-23

**Authors:** Susan E Ivie, Mark S McClain, Holly M Scott Algood, D Borden Lacy, Timothy L Cover

**Affiliations:** 1 Department of Microbiology and Immunology, Vanderbilt University School of Medicine, Nashville, TN 37232, USA; 2 Department of Medicine, Vanderbilt University School of Medicine, Nashville, TN 37232, USA; 3 Veterans Affairs Tennessee Valley Healthcare System, Nashville, TN 37212, USA

## Abstract

**Background:**

*Helicobacter pylori *is a gram-negative bacterium that colonizes the human stomach and contributes to the development of gastric cancer and peptic ulcer disease. VacA, a toxin secreted by *H. pylori*, is comprised of two domains, designated p33 and p55. Analysis of the crystal structure of the p55 domain indicated that its structure is predominantly a right-handed parallel β-helix, which is a characteristic of autotransporter passenger domains. Substitution mutations of specific amino acids within the p33 domain abrogate VacA activity, but thus far, it has been difficult to identify small inactivating mutations within the p55 domain. Therefore, we hypothesized that large portions of the p55 domain might be non-essential for vacuolating toxin activity. To test this hypothesis, we introduced eight deletion mutations (each corresponding to a single coil within a β-helical segment spanning VacA amino acids 433-628) into the *H. pylori *chromosomal *vacA *gene.

**Results:**

All eight of the mutant VacA proteins were expressed by the corresponding *H. pylori *mutant strains and underwent proteolytic processing to yield ~85 kDa passenger domains. Three mutant proteins (VacA Δ484-504, Δ511-536, and Δ517-544) were secreted and induced vacuolation of mammalian cells, which indicated that these β-helical coils were dispensable for vacuolating toxin activity. One mutant protein (VacA Δ433-461) exhibited reduced vacuolating toxin activity compared to wild-type VacA. Other mutant proteins, including those containing deletions near the carboxy-terminal end of the β-helical region (amino acids Val^559^-Asn^628^), exhibited marked defects in secretion and increased susceptibility to proteolytic cleavage by trypsin, which suggested that these proteins were misfolded.

**Conclusions:**

These results indicate that within the β-helical segment of the VacA p55 domain, there are regions of plasticity that tolerate alterations without detrimental effects on protein secretion or activity, as well as a carboxy-terminal region in which similar alterations result in protein misfolding and impaired secretion. We propose that non-essential β-helical coils and a carboxy-terminal β-helical segment required for proper protein folding and secretion are features shared by numerous autotransporter passenger domains.

## Background

Numerous bacterial pathogens secrete virulence factors by a type V (autotransporter) pathway [1]. Crystallographic studies of three passenger domains secreted by a classical (type Va) autotransporter pathway revealed that each has a predominantly β-helical structure [2-4], and it is predicted that nearly all autotransporter passenger domains share a β-helical fold [5]. Very little is known about the structural features that are responsible for the unique properties of individual autotransporter passenger domains.

The *Helicobacter pylori *VacA toxin is one of the most extensively studied bacterial proteins secreted by a classical autotransporter pathway [6-9]. VacA is classified as a pore-forming toxin, but unlike many other bacterial pore-forming toxins, VacA is internalized by cells and can cause cellular alterations by acting intracellularly [6,7,10]. VacA causes a wide array of alterations in mammalian cells, including cell vacuolation, mitochondrial alterations, and plasma membrane permeabilization [6,8], and targets a variety of cell types, including gastric epithelial cells [11], T cells [12,13], and mast cells [14,15]. Several lines of evidence suggest that VacA contributes to the development of *H. pylori*-associated peptic ulcer disease and gastric adenocarcinoma in humans [6,11,16-18].

**Figure 1:**
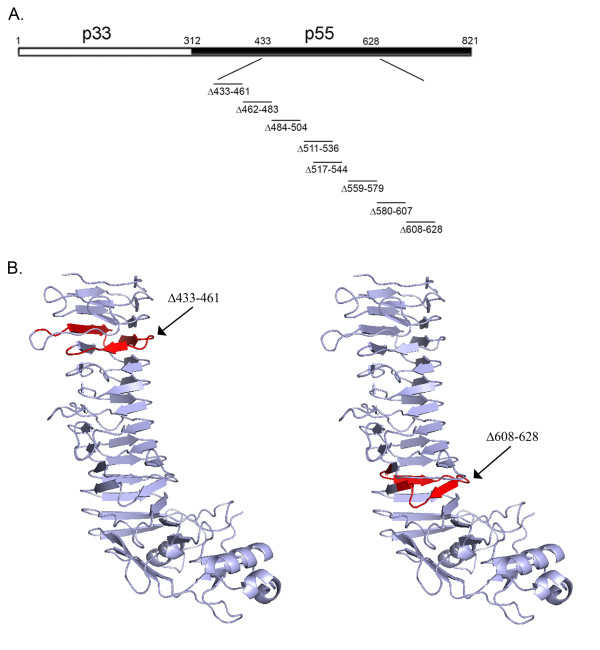
**Introduction of deletion mutations into the VacA p55 domain**. (A) Diagram of the full-length 88 kDa VacA protein secreted by *H. pylori *strain 60190 [19]. p33 (amino acids 1 to 311) and p55 (amino acids 312-821) domains are shown. Mutations encoding single coil deletions within the β-helix of the p55 domain were introduced into the *H. pylori *chromosomal *vacA *gene by natural transformation and allelic exchange as described in Methods. The relative position of each single coil deletion is shown. (B) Crystal structure of the p55 VacA domain of *H. pylori *strain 60190 [3]. The sites of two coils targeted for deletion mutagenesis (amino acids 433-461 and 608-628) are highlighted in red.

VacA is synthesized as a 140 kDa precursor protein, which undergoes proteolytic processing to yield a 33-amino acid signal sequence, a mature 88 kDa secreted toxin, a ~12 kDa secreted peptide, and a carboxy-terminal domain that remains associated with the bacteria [18-20]. The mature 88 kDa VacA passenger domain can be proteolytically processed into an amino-terminal 33 kDa (p33) fragment and a carboxy-terminal 55 kDa (p55) fragment [21], which are considered to represent two domains or subunits of VacA [18,22,23] (Fig. 1A). When expressed intracellularly in eukaryotic cells, about 422 residues at the amino-terminus of VacA (comprising the p33 domain and part of the p55 domain) are sufficient to cause cell vacuolation [24]. Previous studies have shown that the amino-terminal hydrophobic portion of the p33 domain has an important role in membrane channel formation [24-27]. Components of both the p33 domain and the p55 domain are required for VacA oligomerization [3,28,29], and components of the p55 domain are required for VacA binding to host cells [22,30,31].

Recently the crystal structure of the p55 domain of a VacA protein was determined [3]. The most striking feature of this domain is the presence of a right-handed parallel β-helical structure, composed of coiled, parallel β-sheet structures (Fig. 1B). Each coil of the parallel β-helix consists of three parallel β-strands connected by loops of different lengths. The β-helical portion of the VacA p55 domain of *H. pylori *strain 60190 consists of about 13 coils (Fig. 1B) [3]. Substitution mutagenesis of single amino acids within the amino-terminal region of the p33 domain is sufficient to ablate multiple activities of VacA [24-27], but in contrast, it has been difficult to identify small inactivating mutations within the p55 domain [26]. The only known small inactivating mutation within the p55 domain is a deletion of two amino acids (aspartic acid 346 and glycine 347, located in a region of the p55 domain not included in the crystal structure) [29,32], which results in defective oligomerization of VacA.

Since it has been difficult to identify small inactivating mutations within the p55 domain [26], we hypothesized that large portions of the p55 domain might be non-essential for vacuolating toxin activity. To test this hypothesis, in the current study we generated a set of *H. pylori *mutant strains expressing VacA proteins in which individual coils of the p55 β-helix were deleted, and we then analyzed the secretion and activity of these mutant proteins. We report that within the VacA β-helix, there are regions of plasticity that tolerate alterations without detrimental effects on protein secretion or activity, as well as a carboxy-terminal region in which similar alterations result in impaired secretion and protein misfolding.

## Methods

### *H. pylori *strains and growth conditions

*H. pylori *wild-type strain 60190 (ATCC 49503) was the parent strain used for construction of all mutants in this study. The sequence of the VacA protein encoded by this strain is deposited as GenBank accession number Q48245. Throughout this study, we use an amino acid numbering system in which residue 1 refers to alanine 1 of the secreted 88 kDa VacA protein, and the p55 domain corresponds to amino acids 312 to 821. *H. pylori *strains were grown on trypticase soy agar plates containing 5% sheep blood at 37°C in ambient air containing 5% CO_2_. *H. pylori *liquid cultures were grown in sulfite-free Brucella broth containing 10% fetal bovine serum (BB-FBS).

### Introduction of deletion mutations into the chromosomal *vacA *gene of *H. pylori*

To introduce in-frame internal deletion mutations into a plasmid encoding VacA, we performed inverse PCR using pMM592 (encoding wild-type VacA, amino acids 1 to 821) [33] as template DNA, 5'-phosphorylated primers, and Pfu Turbo polymerase (Stratagene). The resulting PCR products were then ligated and transformed into *E. coli *DH5α. Each plasmid was analyzed by DNA sequencing to verify that the desired deletion was present. To introduce the mutations into the *H. pylori *chromosomal *vacA *gene [25,34,35], *H. pylori *strains containing a *sacB*-kanamycin cassette within *vacA *[36] were transformed with plasmids containing *vacA *deletion mutations. Three strains (VM025, VM018, and VM028), each derived from *H. pylori *strain 60190 and each containing the *sacB*-kanamycin cassette in a different site within *vacA *[36], were used to facilitate construction of the desired mutants. Sucrose-resistant, kanamycin-sensitive transformants were selected by growth on Brucella broth plates supplemented with 10% FBS and 5.5% sucrose [36]. Full-length *vacA *sequences encoding the secreted p88 VacA protein were PCR-amplified from mutant strains, and the nucleotide sequences of PCR products were analyzed to confirm that the desired mutation had been introduced successfully into the chromosomal *vacA *gene.

### Immunoblot analysis of VacA

To detect VacA expression, proteins in individual samples were separated by SDS-polyacrylamide gel electrophoresis, transferred to nitrocellulose membrane, and immunoblotted using a polyclonal rabbit anti-VacA antiserum (#958) raised against the secreted 88 kDa passenger domain [37], followed by horseradish peroxidase-labeled rabbit IgG. Peptide mapping experiments, using a set of overlapping 16-amino-acid peptides derived from VacA, indicate that the polyclonal anti-VacA antiserum #958 reacts with at least 10 different epitopes distributed throughout the secreted 88 kDa VacA protein, including the amino-terminus (amino acids 1-16) and the carboxy-terminus (amino acids 813-828) (our unpublished data). To confirm similar loading of lysates from wild-type and mutant *H. pylori *strains, the lysates were immunoblotted with rabbit antiserum to HspB (a GroEL heat shock protein homolog) [38]. The anti-HspB serum was also used to detect the potential release of HspB into culture supernatant by autolysis. Signals were generated by the enhanced chemiluminescence reaction and detected using x-ray film.

### Preparation of broth culture supernatants and normalization of VacA concentrations

*H. pylori *strains were grown in BB-FBS for 48 hours. Broth culture supernatants were concentrated 30-fold by ultrafiltration with a 30 kDa cutoff membrane. Relative concentrations of VacA in different broth culture supernatant preparations were determined by antigen-detection ELISA [36]. Broth culture supernatants were diluted in carbonate buffer (18 mM Na_2_CO_3_, 34.8 mM NaHCO_3_) and allowed to adhere to an ELISA plate overnight at room temperature. After removal of unbound VacA proteins, wells were blocked with phosphate buffered saline (PBS) containing 3% BSA and 0.05% Tween 20. VacA was detected with rabbit anti-VacA antiserum (#958) and horseradish peroxidase-labeled rabbit IgG followed by TMB substrate (Pierce). To permit normalization of VacA concentrations in different preparations, samples were diluted with appropriate quantities of culture supernatant from a *vacA *null mutant strain, based on the antigen-detection ELISA results.

### Sonication of *H. pylori*

*H. pylori *grown on blood agar plates were suspended in sonication buffer [20 mM Tris-acetate (pH 7.9), 50 mM potassium acetate, 5 mM Na_2_EDTA, 1 mM dithiothreitol (DTT), protease inhibitor cocktail] and sonicated on ice for three 10 second pulses. The lysate was centrifuged at 15,000 rpm and the supernatant collected.

### Susceptibility of VacA to proteolysis by trypsin

*H. pylori *grown on blood agar plates were suspended in phosphate buffered saline (PBS), and bacterial suspensions were treated with trypsin (0.05%) for 30 min at 37°C. After addition of a protease inhibitor cocktail, the bacteria were pelleted, and the pellet washed once with PBS containing protease inhibitor. The pellet was then suspended in SDS lysis buffer, boiled, and analyzed by immunoblot. Sonicated preparations of *H. pylori *were treated with trypsin and analyzed in the same manner.

### Analysis of VacA reactivity with a monoclonal antibody

Concentrated culture supernatants containing different VacA mutant proteins were adjusted so that the VacA concentrations were normalized, and then were diluted in carbonate buffer and allowed to adhere to an ELISA plate overnight at room temperature. After removal of unbound VacA proteins, wells were blocked with phosphate buffered saline (PBS) containing 3% BSA and 0.05% Tween 20. VacA was detected with mouse anti-VacA (5E4) [35] and horseradish peroxidase-labeled mouse IgG followed by TMB substrate (Pierce).

### Cell culture analysis of VacA proteins

HeLa cells were grown as described previously [22]. AZ-521 cells (a human gastric adenocarcinoma cell line, Culture Collection of Health Science Research Resources Bank, Japan Health Sciences Foundation) and RK13 cells (ATCC CCL-37, a rabbit kidney cell line) were grown in minimal essential medium supplemented with 10% FBS and 1 mM non-essential amino acids. For vacuolating assays, cells were seeded at 2 × 10^4 ^cells/well into 96-well plates 24 hours prior to each experiment. The VacA content of different samples was normalized as described above. Serial dilutions of samples were added to serum-free tissue culture medium overlying cells (supplemented with 5 mM ammonium chloride) and incubated for 8-10 hours at 37°C. An equivalent volume of a corresponding preparation from a *vacA *null mutant was used as a negative control. After incubation, cell vacuolation was examined by inverted light microscopy and quantified by a neutral red uptake assay [39]. Neutral red uptake data are presented as A_540 _values (mean ± S.D.). Background levels of neutral red uptake by cells treated with culture supernatant from a *vacA *null mutant were subtracted to yield net neutral red uptake values.

## Results

### Expression and secretion of mutant VacA proteins by *H. pylori*

The structure of the VacA p55 domain is dominated by β-helical coils [3]. In previous studies, it has been difficult to identify specific amino acids within the p55 domain that are important for toxin activity [26]. To determine whether specific β-helical elements within the VacA p55 domain are required for VacA activity, we introduced an ordered series of eight deletion mutations, each 20 to 28 amino acids in length, into a portion of the *vacA *gene that encodes the p55 domain. These deletion mutations were designed so that each would result in the deletion of a single coil of the β-helix (Fig. 1A; representative single coils are highlighted in Fig. 1B). By designing the deletion mutations in this manner, it was predicted that the mutant proteins would exhibit reductions in the length of the β-helical region but would exhibit minimal changes in protein folding in comparison to the wild-type VacA protein. All of the deletion mutations analyzed in this study are located outside of the VacA region (amino acids 1-422) previously found to be required for cell vacuolation when VacA is expressed in transiently transfected cells [24]. Each of the mutations was introduced into the *H. pylori *chromosomal *vacA *gene by natural transformation and allelic exchange as described in Methods.

**Figure 2:**
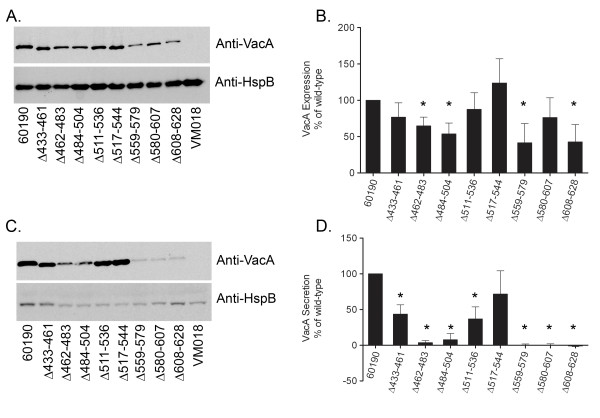
**Expression and secretion of wild-type and mutant VacA proteins**. *H. pylori *wild- type strain 60190, strains expressing mutant forms of VacA, and a *vacA *null mutant strain (VM018) [36] were grown in broth culture. Broth cultures were normalized by optical density (OD 600 nm) and then pellets (A) and unconcentrated broth culture supernatants (C) were analyzed by immunoblot assay using polyclonal anti-VacA serum #958. Samples were also immunoblotted with a control antiserum against *H. pylori *heat shock protein (HspB). The intensity of immunoreactive VacA bands was quantified by densitometry (panels B and D). Wild-type VacA and each of the mutant proteins were expressed and proteolytically processed to yield ~85-88 kDa proteins that were secreted into the broth culture supernatant. Western blots depict representative results from one of three independent experiments; histograms represent results pooled from three independent experiments. Results represent the mean ± SD. *, p < 0.05 compared to wild-type VacA, as determined by Student's t-test.

Each mutant *H. pylori *strain was tested by immunoblot analysis for the capacity to express VacA. We first analyzed expression of the mutant strains grown on blood agar plates. Each mutant strain expressed a VacA protein with a mass of ~85 kDa (corresponding to the VacA passenger domain), which indicated that in each case, the ~140 kDa VacA protoxin underwent proteolytic processing similar to wild-type VacA (data not shown). We next analyzed expression and secretion of VacA when the bacteria were grown in broth culture. Wild-type *H. pylori *and each of the mutant strains exhibited similar patterns of growth. Immunoblot analysis of the bacterial cell pellets indicated that, as expected, each of the mutant strains expressed an ~85 kDa VacA protein (Fig. 2A). In comparison to wild-type VacA, several of the mutant VacA proteins were present in reduced amounts in the bacterial cell pellets (Fig. 2A and 2B). Immunoblot analysis of the broth culture supernatants indicated that each of the mutant strains secreted or released an ~85 kDa VacA protein. In comparison to secretion of VacA by the wild-type strain, several of the mutant VacA proteins were secreted at moderately reduced levels, and three mutant proteins (VacA Δ559-579, Δ580-607, and Δ608-628) were nearly undetectable in culture supernatant (Fig. 2C and 2D). Analysis of the culture supernatants by ELISA yielded similar results (data not shown). Thus, all eight of the mutant proteins were expressed and underwent proteolytic processing similar to that of wild-type VacA, but there was substantial variation among the mutant proteins in the levels of expression and secretion.

### Susceptibility of VacA mutant proteins to proteolytic cleavage by trypsin

**Figure 3:**
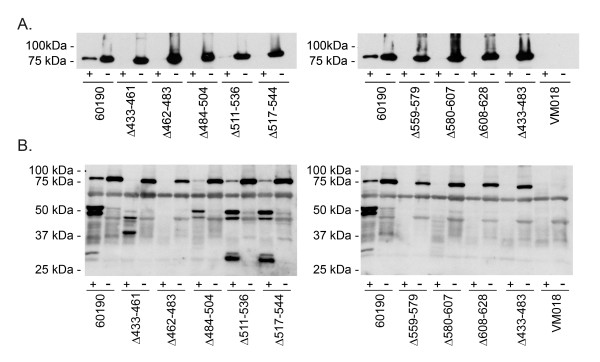
**Susceptibility of VacA proteins to proteolytic cleavage by trypsin**. A) Intact *H. pylori *strains [wild-type strain 60190, strains expressing mutant forms of VacA, and a *vacA *null mutant strain (VM018)] were suspended in PBS and incubated in the presence (+) or absence (-) of trypsin as described in Methods. After centrifugation, bacterial pellets were analyzed by immunoblot analysis using polyclonal anti-VacA serum #958. (B) *H. pylori *strains were sonicated as described in Methods. After centrifugation, the soluble fractions were analyzed further. The total protein concentration of each sample was approximately 7.5 μg/ml, as determined by A_280 _values (data not shown). Samples were incubated in the presence (+) or absence (-) of trypsin and analyzed by immunoblot analysis using polyclonal anti-VacA serum #958.

Previous studies have shown that the wild-type 88 kDa VacA passenger domain is secreted and released into the extracellular space and that 88 kDa proteins also remain localized on the surface of *H. pylori *[40]. To investigate whether the mutant VacA proteins were able to localize on the bacterial surface similar to wild-type VacA, the wild-type and mutant *H. pylori *strains were harvested from blood agar plates and treated with trypsin as described in Methods. Trypsin is expected to proteolytically cleave proteins on the surface of the bacteria, but not intracellular proteins [7]. Each of the ~85 kDa mutant proteins was cleaved by trypsin (Fig. 3A), which provided evidence that these mutant VacA proteins are transported across the inner and outer membranes and localize on the surface of the bacteria.

To analyze potential differences in folding properties of the VacA mutant proteins compared to wild-type VacA, we analyzed the susceptibility of these proteins to proteolytic cleavage. Lysates of *H. pylori *strains were generated by sonication, and the solubilized proteins were treated with trypsin as described in Methods. Trypsin digestion of two of the mutant proteins (Δ511-536 and Δ517-544) yielded proteolytic digest patterns that were identical to each other and similar to that of trypsin-digested wild-type VacA (Fig. 3B). Trypsin digestion of two other mutant proteins (Δ433-461 and Δ484-504) yielded different digest patterns, but these mutant proteins were not completely degraded (Fig. 3B). Four mutant proteins (Δ462-483, Δ559-579, Δ580-607, and Δ608-628) were completely degraded by trypsin (Fig. 3B). In general, the four mutant proteins that exhibited relative resistance to trypsin digestion were secreted at relatively high levels compared to mutant proteins that were completely degraded by trypsin (compare Fig. 2 and Fig. 3B). The observed variation among mutant VacA proteins in susceptibility to trypsin-mediated proteolysis suggested that the individual mutant proteins differed in folding properties. The proteins that were highly susceptible to trypsin digestion and secreted at very low levels (Δ462-483, Δ559-579, Δ580-607, and Δ608-628) were probably misfolded. Due to the very low concentrations of these four proteins in the broth culture supernatants, these mutant VacA proteins were not studied further.

**Figure 4:**
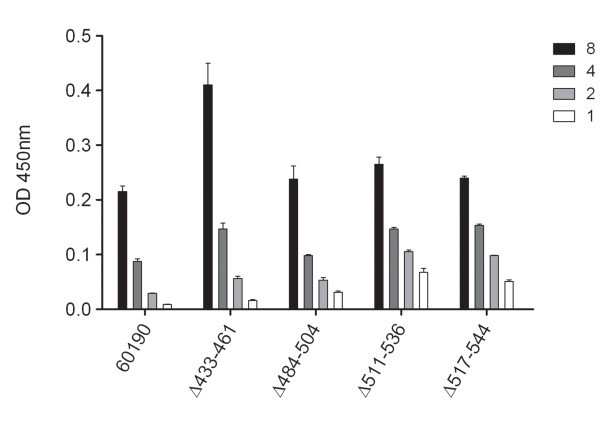
**Reactivity of VacA mutant proteins with a monoclonal anti-VacA antibody**. Wild-type *H. pylori *strain 60190 and strains expressing mutant VacA proteins were grown in broth culture, and secreted VacA proteins were normalized as described in Methods. Wells of ELISA plates were coated with broth culture supernatants, and reactivity of the proteins with an anti-VacA monoclonal antibody (5E4) that recognizes a conformational epitope was determined by ELISA. Reactivity of a *vacA *null mutant was subtracted as background. Relative VacA concentrations are indicated. Values represent the mean ± SD from triplicate samples.

To evaluate whether the four mutant proteins exhibiting relative resistance to trypsin-mediated proteolysis (i.e. VacA Δ433-461, Δ484-504, Δ511-536, and Δ517-544) shared other features with wild-type VacA, we analyzed the reactivity of these proteins with an anti-VacA monoclonal antibody (5E4) that recognizes a conformational epitope [35]. Each of the four mutant VacA proteins was recognized by the 5E4 antibody (Fig. 4), which provided additional evidence that these mutant proteins were folded in a manner similar to that of wild-type VacA.

### Analysis of vacuolating activity of mutant VacA proteins

We next investigated whether the mutant VacA proteins retained vacuolating toxin activity. As a first approach, we attempted to purify the mutant VacA proteins from *H. pylori *broth culture supernatants, using methods that are well-established for purification of water-soluble oligomeric forms of wild-type VacA or mutant VacA proteins that contain alterations in the p33 domain [26,34,36]. We focused these purification efforts on the four mutant proteins that were secreted at the highest levels and that exhibited evidence of protein folding similar to that of wild-type VacA (i.e. VacA Δ433-461, Δ484-504, Δ511-536, and Δ517-544). The yields of purified mutant proteins were markedly lower than yields of purified wild-type VacA, and several of the VacA mutant proteins were not successfully purified. These results could be attributable to relative defects in oligomerization of mutant proteins compared to wild-type VacA, or could be attributable to other altered properties of the mutant proteins that resulted in aberrant behavior during the purification procedure.

**Figure 5:**
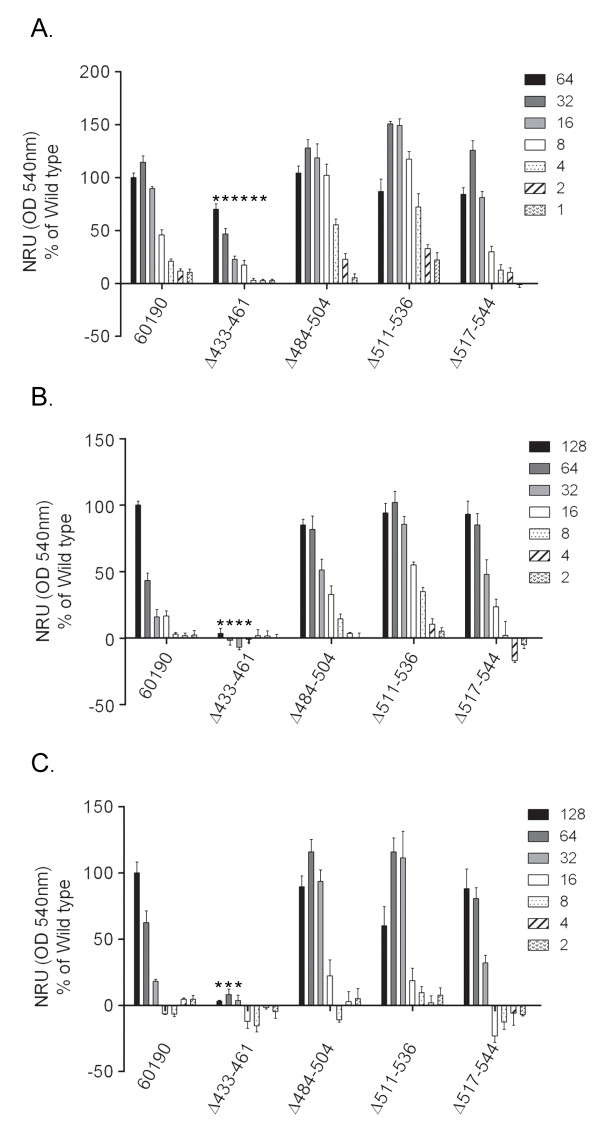
**Vacuolating cytotoxic activity of mutant proteins**. Wild-type *H. pylori *strain 60190 and strains expressing mutant VacA proteins were grown in broth culture, and secreted VacA proteins were normalized as described in Methods. Serial two-fold dilutions of VacA-containing preparations were added to HeLa cells (A), RK13 cells (B), and AZ-521 cells (C). Vacuolating activity was measured by neutral red uptake. Relative VacA concentrations are indicated. Results represent the mean ± SD from triplicate samples, expressed as a percent of neutral red uptake induced by wild-type VacA. *, p ≤ 0.02 as determined by Student's t-test compared to wild type VacA. Similar results were observed in three independent experiments.

Since it was not possible to purify sufficient quantities of the mutant VacA proteins to permit analysis of vacuolating toxin activity, we used an alternative approach. *H. pylori *culture supernatants containing wild-type VacA or mutant proteins were normalized by ELISA so that the VacA concentrations were similar, as described in Methods, and then were tested for vacuolating toxin activity. Using this approach, it was possible to test the activity of the four mutant proteins that were secreted at the highest levels and that exhibited evidence of protein folding similar to that of wild-type VacA (i.e. VacA Δ433-461, Δ484-504, Δ511-536, and Δ517-544), but analysis of the remaining VacA mutant proteins (which exhibited evidence of defective folding) was not possible due to prohibitively low concentrations of the secreted mutant proteins and inability to normalize the concentrations of these proteins. The mutant proteins were initially tested for ability to induce vacuolation of HeLa cells, a cell line that is commonly used for the study of VacA activity. Each of the mutant proteins (VacA Δ433-461, Δ484-504, Δ511-536, and Δ517-544) induced vacuolation of HeLa cells (Fig. 5A), but one of the mutants, VacA Δ433-461, exhibited reduced vacuolating activity compared to wild-type VacA. The same preparations of mutant proteins were then tested for their ability to induce vacuolation of AZ-521 cells (human gastric epithelial cells) and RK13 cells (rabbit kidney cells), two cells lines that have been used for analysis of VacA activity [41-43]. VacA Δ484-504, Δ511-536, and Δ517-544 each caused vacuolation of RK13 and AZ-521 cells, but VacA Δ433-461 lacked detectable vacuolating activity for both RK13 and AZ-521 cells (Fig. 5B and 5C). Thus, three of mutant proteins caused vacuolation of all the tested cell lines in a manner similar to wild-type VacA, whereas VacA Δ433-461 caused reduced vacuolation of HeLa cells and did not cause detectable vacuolation of RK13 or AZ-521 cells. These data suggest that residues within the region spanning amino acids 433 to 461 contribute to VacA activity, and indicate that the β-helical coils corresponding to amino acids 484-504, 511-536 and 517-544 are dispensable for vacuolating toxin activity.

## Discussion

In this study, we sought to identify regions of the p55 β-helix that are either essential or non-essential for vacuolating toxin activity. All of the VacA mutant proteins analyzed in this study were designed in a manner that resulted in the deletion of a single coil of the β-helix, based on analysis of the crystal structure of the VacA p55 domain [3]. We predicted that all of the mutant VacA proteins would retain a β-helical structure, and that this mutagenesis approach would result in minimal disruptions in protein folding.

As a first step, we analyzed the proteolytic processing and secretion of the mutant proteins. All eight of the mutant VacA proteins were expressed by the corresponding *H. pylori *mutant strains and underwent proteolytic processing to yield ~85 kDa passenger domains. We found that several individual coils within the p55 domain could be deleted without substantially altering the capacity of the proteins to undergo secretion by *H. pylori*. In contrast, the deletion of other coils led to a marked defect in VacA secretion. The mutant proteins that exhibited marked defects in secretion also exhibited increased susceptibility to proteolytic cleavage by trypsin, which suggested that these proteins were misfolded. In addition to the mutant VacA proteins shown in Figure 1, we also generated *H. pylori *mutant strains expressing VacA proteins in which two coils (Δ433-483) or four coils (Δ433-529) of the β-helix were deleted. These mutant strains expressed truncated VacA proteins of the expected size (approximately 82 and 77 kDa, respectively) at levels similar to the level of wild-type VacA expression, but these mutant proteins were poorly secreted (data not shown). These findings suggest that VacA proteins containing large deletions (more than one coil) within the β-helical region of the p55 domain are poorly secreted. Similarly, a previous study reported efforts to introduce large deletions into the region of the *H. pylori *chromosomal *vacA *gene that encodes the VacA p55 domain, and most of the resulting mutant proteins were neither expressed nor secreted by *H. pylori *[36].

In the current study, the three mutant VacA proteins that exhibited the most striking defects in secretion (Δ559-579, Δ580-607, Δ608-628) each contained deletions that are localized near the carboxy-terminus of the β-helix. Interestingly, a study of *Bordetella pertussis *BrkA revealed that a β-helical region near the carboxy-terminus of the passenger domain is required for folding of this protein [44]. The authors proposed that this domain acts as an intramolecular chaperone to promote folding of the passenger domain concurrent with or following translocation through the outer membrane. Similarly, studies of *B. pertussis *pertactin indicate that the carboxy-terminal β-helical region of this protein exhibits enhanced stability and can fold as a stable core structure [5,45]. We speculate that VacA amino acids 559-628 have a similar functional role in promoting protein folding and secretion.

An important finding in the current study is that, within the VacA β-helix, there are regions of plasticity that tolerate alterations without detrimental effects on protein secretion or toxin activity. VacA Δ484-504, Δ511-536, and Δ517-544 mutant proteins each retained vacuolating activity similar to that of wild-type VacA, which indicates that the corresponding coils are dispensable for vacuolating toxin activity. The retention of vacuolating activity despite the deletion of entire coils of the β-helix correlates well with results from a previous study, which reported that inactivating point mutations within the portion of *vacA *encoding the p55 domain could not be identified [26]. One of the VacA mutant proteins analyzed in the current study (Δ433-461) exhibited detectable vacuolating toxin activity on HeLa cells, but its activity on HeLa cells was reduced compared to that of wild-type VacA, and it lacked detectable activity on RK13 cells and AZ521 cells. These data suggest that residues within this VacA region (amino acids 433-461) have an important role in VacA activity. Further studies may lead to the identification of specific amino acids within this region that mediate interactions between VacA and host cells. Similar to most previous studies, the current study assessed the effects of VacA mutations on the ability of VacA to cause cell vacuolation. Future investigations may provide new insights into structural properties of VacA that are required for other actions of this multifunctional toxin.

## Conclusions

VacA is a unique toxin that is considered to be an important determinant of *H. pylori *virulence, and therefore, it is important to have an in-depth understanding of VacA structure and function. The VacA p55 structure is predominantly a right-handed parallel β-helix, which is a characteristic of autotransporter passenger domains. Previous studies suggested that the p55 domain contributes to VacA binding to cells, but it has been difficult to identify small inactivating mutations within the p55 domain. Therefore, we hypothesized that large segments of the p55 domain might be non-essential for vacuolating toxin activity. To test this hypothesis, we constructed and analyzed a set of *H. pylori *mutant strains expressing VacA proteins in which individual coils of the beta-helix were deleted. Three mutant proteins containing deletions in the region spanning VacA amino acids 484-544 were efficiently secreted and induced vacuolation of mammalian cells, which indicates that these segments are dispensable for vacuolating toxin activity. We also identified a region near the carboxy-terminal end of the β-helix (amino acids 559-628), in which the introduction of similar deletion mutations resulted in marked defects in protein secretion and apparent defects in protein folding. We propose that non-essential β-helical coils and a carboxy-terminal β-helical segment required for proper protein folding and secretion are features of numerous autotransporter passenger domains.

## Authors' contributions

Conceived and designed the experiments: SEI, MSM, DBL, TLC. Performed the experiments: SEI. Analyzed the data: SEI, MSM, HMSA, DBL, TLC. Wrote the paper: SEI, TLC. All authors read and approved the final manuscript.
